# The Effect of Adding Mountain Mint (*Ziziphora clinopodioides* Lam.) to Diet of Laying Japanese Quails on Productive Performance, Egg Quality Traits, Blood Metabolites and Immune Response

**DOI:** 10.1002/vms3.70945

**Published:** 2026-04-24

**Authors:** Ahmad Hassanabadi, Mojtaba Teymoori Oghaz, Heydar Zarghi

**Affiliations:** ^1^ Department of Animal Science Faculty of Agriculture Ferdowsi University of Mashhad Mashhad Iran

**Keywords:** blood metabolites, egg quality, immune response, Japanese quail, mountain mint, performance

## Abstract

**Background:**

Mountain mint (MM), a traditional herbal remedy, has garnered attention for its potential benefits on poultry health and productivity.

**Objectives:**

This experiment aimed to evaluate the effects of dietary supplementation with MM on the immune response, productive performance, egg quality traits and blood metabolites of laying Japanese quails.

**Methods:**

A total of 375 quails, aged 6 weeks, were used in a 14‐week trial in a completely randomized design with 5 treatments and 5 replicates of 15 birds each. The treatments included five levels of MM powder: 0% (control), 0.2%, 0.4%, 0.6% and 0.8% of the diet.

**Results:**

Adding MM to the diet had no significant effects on egg production, egg mass and weight, feed intake or feed conversion ratio. Increasing MM levels significantly decreased egg‐shape index (*p* < 0.05). The highest egg‐shape index was observed in control and 0.2% MM groups (79.9%), whereas the lowest was observed in 0.8% treatment (75.1%). MM significantly increased blood high‐density lipoprotein cholesterol (HDL‐C) and decreased low‐density lipoprotein cholesterol (LDL‐C) concentrations (*p* < 0.05). Highest HDL‐C concentration was observed in 0.8% MM, which was significantly (*p* < 0.05) higher than that in the control and other treatments. Lowest LDL‐C was observed in 0.4%–0.8% MM, which was significantly (*p* < 0.05) lower than that of control and 0.2% MM. MM linearly decreased LDL‐C (*p* < 0.05). Dietary MM improved primary immune responses, as evidenced by higher immunoglobulin M (IgM) titres, whereas immunoglobulin G (IgG) and total anti‐sheep red blood cells (SRBC) titres remained unchanged.

**Conclusions:**

Adding MM to the diet had no significant effect on productive performance, resulted in the production of elongated eggs and improved blood lipid profiles and primary immune responses in laying quails.

## Graphical abstract

Adding mountain mint (*Ziziphora clinopodioides* Lam.) to diet of laying quails resulted in the production of elongated eggs, improved blood lipid profiles and immune responses.

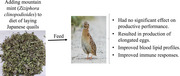

## 1 Introduction

The quest to enhance poultry health and productivity has led researchers to explore various natural feed additives, including herbs with potential nutritional and therapeutic benefits. The use of phytogenic feed additives derived from plants, especially medicinal and aromatic plants containing biologically active substances, is increasing due to health and diversity concerns. These plant‐derived additives contain bioactive compounds that may positively influence poultry performance and physiological status. Since the beginning of the 21st century, considerably more studies have been conducted on the use of medicinal plants or their derivatives in animal feed than in previous decades (Abd El‐Hack et al. 2025).

Among the mint family, several useful and valuable species exist, some of which are utilized for essential oil production. The essential oils of plants belonging to this family are alkaloid‐free (Nabizadeh Asl and Valizadeh 2017). The most common essential oils from the mint family include mint, thyme, rosemary and lavender. These essential oils contain aromatic compounds that, in addition to their pleasant aroma and flavour, possess strong antimicrobial properties. In most pharmacopoeias, among mint species, mountain mint (MM) is noted for its numerous medicinal effects due to its menthol compounds, and its essential oil exhibits antimicrobial activity. To the best of the authors’ knowledge, limited information is available regarding the effects of MM on the performance of Japanese quails. However, its bioactive compounds may positively influence productive performance and egg quality through antioxidant, antimicrobial and metabolic modulation mechanisms (Jaderi et al. 2011; Sharifi‐Rad et al. 2017).

MM is a plant from the mint family that exhibits antimicrobial effects. The essential oil of MM contains pulegone as its most common component, and its beneficial effects on *Saccharomyces* yeast are well established (Nabizadeh Asl and Valizadeh 2017). The primary components of MM essential oil have been reported as pulegone (65.2%), isomenthone (11.9%), cineole (7.8%) and piperitenone (6.5%). Although essential oil compositions of similar subspecies are generally comparable, variations in pulegone content (50%–60%) have been reported, indicating the influence of environmental and laboratory conditions on essential oil composition (Nabizadeh Asl and Valizadeh 2017).

Blood metabolites are critical indicators of physiological status and nutritional adequacy in animals. These include glucose, cholesterol and proteins, which play essential roles in energy metabolism and overall health. In laying quails, maintaining optimal blood metabolite status particularly lipid transport indicators such as lipoprotein fractions is important for reproductive performance and egg quality, as these parameters reflect metabolic efficiency and nutrient partitioning (Alagawany et al. 2021). Furthermore, humoral immune responses, characterized by antibody production in response to pathogens, are vital for disease protection and longevity. Enhancing immune responses through dietary interventions can improve health outcomes and reduce susceptibility to infections (Mehrzad et al. 2024).

Although phytogenic feed additives have been shown to influence blood metabolites and immune responses in poultry, experimental data regarding the effects of MM on blood lipid profile and humoral immune response in Japanese quails are limited. Preliminary studies suggest that MM bioactive compounds, such as flavonoids and phenolic acids, may enhance antioxidant activity, reduce oxidative stress and improve metabolic and immune functions (Abd El‐Hack et al. 2025).

Therefore, the aim of this study was to investigate the effects of adding MM plant to the diet on production performance, egg quality traits, blood metabolites and immune response in laying Japanese quails.

## 2 Materials and Methods

### 2.1 Preparation and Chemical and Essential Oil Analysis of MM

Fresh MM plants were purchased from a local vendor and dried in the shade at 24°C for 7 days. All aerial parts of the plant, including leaves and stems, were then powdered using a coffee grinder. The dry matter, crude protein, crude fat, crude fibre, ash, calcium and total phosphorus contents were determined (Table 1) using standard methods (AOAC 2005).

**TABLE 1:** Analysis of the aerial parts of the mountain mint, *as is basis*.

Composition	%
Dry matter	92.7
Crude protein	8.94
Ether extract	3.2
Crude fibre	19.0
Ash	11.7
Nitrogen free extract	82.0
Calcium	0.19
Total phosphorus	0.20

The aerial parts powder was subjected to hydrodistillation using a Clevenger‐type apparatus for 4 h to obtain an essential oil, which was collected in hexane solvent, dried with anhydrous sodium sulphate, weighed and stored at 4°C in the dark until use. The extraction efficiency was 1.9% (w/w), and the relative standard deviation for four extractions was 3.52 parts per trillion (ppt). Analysis of the essential oil was performed using a Hewlett Packard 6890 network GC system equipped with a 60 m × 0.25 mm i.d., 0.25 µm HP‐5 MS capillary column and an HP 5973 mass‐selective detector. Helium was used as the carrier gas at a flow rate of 1 mL/min. Injector and MS transfer line temperatures were set at 250°C and 260°C, respectively. The column temperature was maintained at 40°C for 1 min, then programmed from 40°C to 250°C at a rate of 3°C/min and finally held isothermally for 20 min for GC/MS detection. Electron ionization was carried out at an ionization energy of 70 eV. Retention indices were calculated using the retention times of C8 to C26 *n*‐alkanes injected under the same chromatographic conditions, based on the method of Van den Dool and Kratz (1963). Linear retention indices for all compounds were determined by co‐injection of the sample with a solution containing a homologous series of C8–C26 *n*‐alkanes. Individual constituents were identified by comparing their retention indices with those reported in the literature (Adams 2007) and by matching their mass spectra with those of known compounds or with the Wiley 7 mass spectral database.

### 2.2 Birds, Management and Diets

A total of 375 adult laying quails, aged 6 weeks, were used in this study in a completely randomized design with 5 treatments, 5 replications and 15 quails per replication. Galvanized laying cages with mesh floors measuring 40 × 40 × 37.5 cm^3^ (height × length × width), with a slope of 7.8 degrees, were equipped with a trough feeder at the front of the cages and 2 nipple drinkers. The lighting programme was set to 16 h of light at an intensity of 3.5 W/m^2^ and 8 h of darkness, with the temperature maintained at 21°C ± 2°C. The birds had free access to feed and water throughout the experiment. The experimental period included a 2‐week adaptation phase (from 6 to 8 weeks of age) and a 12‐week recording period (three 28‐day periods) from 9 to 20 weeks of age during the laying phase.

A basal diet was formulated according to the recommended requirements of the National Research Council (NRC, 1994), and the nutrient composition of the feed ingredients is presented in its tables (Table 3). At each level of MM inclusion, cellulose was progressively replaced in the diet. Accordingly, the basal diet (0% MM) contained 0.8% cellulose, whereas the cellulose level was reduced to zero in the diet containing 0.8% MM. Because a single basal formulation was used, the diets were considered isocaloric and isonitrogenous across all treatments. Although MM contained 19.0% crude fibre and cellulose is assumed to be entirely crude fibre, the inclusion rate of MM for cellulose was low (≤0.8% of the diet). Therefore, the resulting change in total dietary fibre was minimal, and the experimental diets were considered nutritionally comparable with respect to crude fibre content. The basal diet was divided into five equal parts. One part was used as the control diet, whereas the other four parts were mixed with 0.2%, 0.4%, 0.6% and 0.8% MM powder, respectively, replacing cellulose either partially or completely.

### 2.3 Productive Performance

Feed intake (FI) was calculated as the difference between the amount of feed remaining at the end of each 28‐day period and the total amount of feed provided during that period. Mortality was recorded daily, and average daily FI was corrected for dead birds. Any feed spilt around the feeder was collected and included in the remaining feed weight. The number and weight of eggs produced in each experimental unit were recorded daily. Performance traits, including egg production, egg weight (EW), egg mass (EM) and feed conversion ratio (FCR), were calculated for each 28‐day period. FCR was calculated by dividing the average daily FI of birds in each replicate by the daily EM produced (g/g).

### 2.4 Egg Quality Traits

To evaluate egg quality parameters, on the last day of the second (16 weeks of age) and third (20 weeks of age) 4‐week periods, four eggs per replicate were randomly selected and transferred to the Egg Quality Laboratory within 4 h of collection. Eggs were weighed using an electronic digital balance (0.001 g, model GF 400; A&D Weighing). Eggs were also weighed while submerged in distilled water to calculate quail egg specific gravity (SG) using the Archimedes method and the following formula:

$$\begin{array}{ccc} & & \mathrm{Egg}\mathrm{specific}\mathrm{gravity}\left(g/cm^{3}\right)=\left(\mathrm{egg}\mathrm{weight}\mathrm{in}\mathrm{air}\right)/\\ & & \left(\mathrm{egg}\mathrm{weight}\mathrm{in}\mathrm{air}\;-\;\mathrm{egg}\mathrm{weight}\mathrm{in}\mathrm{water}\right) \end{array}$$

To measure the egg‐shape index, the length and width of the egg were measured using a digital caliper (0.05 mm, model 1116–150; Insize), and the following formula was used:

$$\mathrm{Shape}\mathrm{index}=((\mathrm{egg}\mathrm{width}\left(\mathrm{mm}\right)/\left(\mathrm{egg}\mathrm{height}\left(\mathrm{mm}\right)\right)\times 100$$

To measure qualitative egg traits, eggs were carefully broken onto a glass plate (35 × 25 cm^2^). The Haugh unit (HU) was calculated using the following formula (Haugh 1937):

$$\mathrm{HU}=100\times \log10[\mathrm{albumenheight}\left(\mathrm{mm}\right)\left(1.7\times \mathrm{eggweight}{\left(g\right)}^{0}.37\right)+7.57]$$

Using a cloth napkin, the albumen residue adhering to the yolk was removed, and then the yolk was weighed. The eggshell was washed with distilled water and placed in a laboratory environment for 48 h to dry before being weighed. The weight of the albumen was calculated by subtracting the weight of the yolk plus the shell from the total EW. The relative weight of the different components of the egg (yolk, white and shell) was calculated by dividing the weight of the egg components by the total EW multiplied by 100. Eggshell thickness (EST) was measured using a micrometre (0.001 mm, model 293‐240; Mitutoyo) at three different points (air sac, equator and sharp end), and the average of these measurements was used to determine the thickness of the eggshell (Alsherify and Hassanabadi 2024).

The yolk colour of quail eggs was visually assessed using the Roche yolk colour fan scale (Roche Ltd., Basel, Switzerland), which ranges from the lightest (Score 1) to the darkest colour (Score 15).

### 2.5 Blood Metabolites

On the final day of the experiment (20 weeks of age), two birds per replicate (10 birds per treatment) were randomly selected, and blood samples were collected from the brachial vein into non‐heparinized tubes. After clotting, samples were centrifuged at 1900 *g* for 5 min at 4°C to separate serum. Serum metabolites, including triglycerides (TGs), total cholesterol (TC), high‐density lipoprotein cholesterol (HDL‐C) and low‐density lipoprotein cholesterol (LDL‐C), were measured using a multi‐assay automated analyser (Cobas Bio, Roche, Basel) (Omary et al. 2024).

### 2.6 Humoral Immune Response

The humoral immune response was evaluated by measuring haemagglutination antibody titres. A 5% suspension of SRBC in phosphate‐buffered saline (PBS) was prepared and stored at 4°C until use. At 14 weeks of age, two birds per replicate were randomly selected, and 0.5 mL of the SRBC suspension was injected into the right breast muscle. Because the primary humoral immune response in poultry typically peaks approximately 7–10 days post‐immunization (Van der Zijpp et al. 1983), 1 mL of blood was collected from the brachial vein 7 days after SRBC injection. Serum was separated and stored at −20°C until antibody determination. Antibody titres were determined by the haemagglutination assay and expressed as log_2_ of the highest serum dilution that agglutinated 0.05 mL of a 2.5% SRBC suspension in PBS (Alsherify and Hassanabadi 2024).

### 2.7 Statistical Analysis

All data were tested for normality using SAS software (version 9.4; Statistical Analysis System [SAS], 2012) with the UNIVARIATE procedure. Non‐normally distributed data were arcsine‐transformed before analysis. Data were analysed using PROC GLM in a completely randomized design. Linear and quadratic responses to increasing dietary MM levels were evaluated using PROC REG. Mean comparisons were performed using Tukey's test at a significance level of *p* < 0.05.

## 3 Results

### 3.1 Essential Oil Analysis of MM

Thirty‐five components were identified in the essential oil extracted from MM; however, only 10 of them are listed with their percentages according to the order of elution, comprising 93.88% of the total oil (Table 2). The major components were pulegone (62.03%), *cis*‐caran‐*trans*‐2‐ol (11.89%), 1,8‐cineole (11.03%) and β‐pinene (2.04%)3.

**TABLE 2:** Major chemical constituents of mountain mint root essential oil identified by gas chromatography.

Number	Compound	Retention index	%
1	α‐Pinene	934	1.39
2	Pulegone	1253	62.03
3	Camphene	948	0.49
4	Sabinene	974	1.44
5	2‐β‐Pinene	976	2.04
6	β‐Myrcene	990	0.92
7	1,8‐cineole	1034	11.03
8	(IR)‐(+)‐*Trans*‐is limonene	1070	1.12
9	(−)*Cis*‐Caran‐*trans*‐2‐ol	1155	11.89
10	Isopulegone	1175	1.53

*Note*: Compounds presented in order of elution from the HP‐5Ms capillary column; retention index relative to *n*‐alkanes on the HP‐5Ms capillary column; percentage based on flame ionization detector (FID) peak area normalization.

**TABLE 3:** Ingredients and nutrients composition of basal diet, *as fed basis*.

Ingredient	%
Corn	57.75
Soybean meal (44% CP)	25.21
Soybean oil	3.40
Limestone	9.82
Dicalcium phosphate	1.89
Common salt	0.41
Vitamin premix^a^	0.25
Mineral premix^b^	0.25
l‐Lysine HCl	0.01
dl‐Methionine	0.17
l‐Threonine	0.04
Cellulose	0.8
**Calculated nutrient composition (%)**	
Metabolizable energy (kcal/kg)	2900
Crude protein	15.5
Calcium	4.2
Available phosphorus	0.48
Sodium	0.18
Digestible methionine	0.40
Digestible met + Cys	0.63
Digestible lysine	0.75
Digestible threonine	0.53

^a^ Vitamin premix supplied the followings per kilogram of diet: vitamin A (all‐*trans*‐retinol), 4400 IU; vitamin D3 (cholecalciferol), 1000 IU; vitamin E (a‐tocopherol), 11 IU; vitamin K3 (menadione), 2.33 mg; vitamin B1 (thiamin), 2.97 mg; vitamin B2 (riboflavin), 4.4 mg; vitamin B3 (niacin), 22 mg; vitamin B5 (pantothenic acid), 10 mg; vitamin B6 (pyridoxine), 4.45 mg; vitamin B9 (folic acid), 1.9 mg; vitamin B12 (cyanocobalamin), 0.011 mg; vitamin H2 (biotin), 0.18 mg; choline chloride, 487.5 mg; and antioxidant, 1.0 mg.

^b^ Mineral premix supplied the followings per kilogram of diet: Zn (zinc oxide), 75 mg; Mn (manganese oxide), 75 mg; Fe (iron sulphate), 75 mg; Cu (copper sulphate), 5 mg; I (ethylene diamine di‐hydroiodide), 0.76 mg; Se (sodium selenite), 0.1 mg; and choline chloride, 474 mg.

### 3.2 Productive Performance

The effect of adding MM aerial parts powder to the diet on egg production percentage, EM and weight in Japanese laying quails at different ages is summarized in Table 4. As shown, adding different levels of MM to the diet had no significant effects on these production parameters. Additionally, adding MM to the diet did not produce linear or quadratic responses in the birds (*p* > 0.05).

**TABLE 4:** Effect of adding mountain mint (*Ziziphora clinopodioides)* to Japanese quails diet on egg production, weight and mass.

Mountain mint (% of diet)	9–12 weeks	13–16 weeks	17–20 weeks
	Egg production (%)		
0 (control)	92.6	77.9	70.8
0.2	90.1	76.8	70.4
0.4	89.7	77.0	70.5
0.6	88.2	78.9	69.0
0.8	88.6	77.5	70.5
SEM	0.658	0.016	0.304
	*p* value		
Treatment	0.603	0.709	0.280
Linear	0.201	0.877	0.571
Quadratic	0.215	0.801	0.658
	Egg mass (g/bird/day)		
0 (control)	12.1	12.4	12.5
0.2	11.7	12.1	11.6
0.4	12.2	12.0	12.1
0.6	11.4	12.1	11.9
0.8	12.0	12.1	12.4
SEM	0.374	0.242	0.116
	*p* value		
Treatment	0.559	0.360	0.853
Linear	0.565	0.402	0.197
Quadratic	0.694	0.478	0.162
	Egg weight (g)		
0 (control)	13.6	13.6	13.7
0.2	13.6	13.7	13.6
0.4	13.5	13.8	13.8
0.6	13.5	13.6	13.5
0.8	13.5	13.6	13.7
SEM	0.101	0.307	0.105
	*p* value		
Treatment	0.868	0.206	0.517
Linear	0.991	0.513	0.547
Quadratic	0.807	0.428	0.614

The effect of adding MM to the diet on FI and TG levels of Japanese laying quails at different ages is summarized in Table 5. As shown, adding MM to the diet had no significant effects on FI and FCR. Moreover, birds fed diets containing different levels of MM did not show linear or quadratic responses (*p* > 0.05).

**TABLE 5:** Effect of adding mountain mint (*Ziziphora clinopodioides)* to Japanese quails diet on feed intake and feed conversion ratio.

Mountain mint (% of diet)	9–12 weeks	13–16 weeks	17–20 weeks
	Feed intake (g/bird/day)		
0 (control)	37.3	39.1	40.1
0.2	37.0	38.3	39.3
0.4	36.7	38.1	39.0
0.6	36.4	37.7	38.7
0.8	38.1	39.4	38.4
SEM	0.771	0.771	0.771
	*p* value		
Treatment	0.600	0.610	0.601
Linear	0.100	0.150	0.931
Quadratic	0.202	0.301	0.916
	Feed conversion ratio (g/g)		
0 (control)	2.79	2.77	2.82
0.2	2.71	2.78	2.88
0.4	2.75	2.82	2.88
0.6	2.76	2.83	2.89
0.8	2.80	2.80	2.89
SEM	0.050	0.049	0.403
	*p* value		
Treatment	0.621	0.633	0.055
Linear	0.969	0.101	0.574
Quadratic	0.401	0.133	0.982

### 3.3 Egg Quality Traits

The effect of dietary MM powder on egg quality traits of Japanese laying quails at 16 weeks of age is presented in Table 6. In this study, adding MM powder to the diet of laying quails caused a significant decrease in egg‐shape index (*p* < 0.05). The highest egg‐shape index was observed in the control group and the 0.2% MM treatment (79.9% in both groups), whereas the lowest value was observed in the 0.8% treatment (75.1%). Additionally, increasing MM powder levels from 0.2% to 0.8% resulted in a significant linear decrease in egg‐shape index (*p* < 0.01); however, no significant quadratic trend was observed (Table 6).

**TABLE 6:** Effect of adding mountain mint (*Ziziphora clinopodioides)* to Japanese quails diet on egg quality traits at 16 weeks of age.

	Mountain mint (% of diet)					SEM	*p* value		
	0 (control)	0.2	0.4	0.6	0.8		Treatment	Linear	Quadratic
Specific gravity (g/cm^3^)	1.06	1.06	1.06	1.05	1.06	0.229	0.302	0.841	0.811
Albumen (%)	54.6	53.8	54.5	53.3	55.3	1.287	0.756	0.472	0.417
Yolk (%)	31.7	33.5	32.5	32.4	30.3	0.972	0.143	0.170	0.079
Shell (%)	13.7	12.7	13.1	14.4	14.3	0.680	0.396	0.482	0.256
Shape index (%)	79.9^a^	79.9^a^	77.1^ab^	76.8^ab^	75.1^b^	1.096	0.021	>0.001	0.364
Haugh unit	81.1	82.5	81.7	80.0	81.0	1.283	0.296	0.237	0.444
Yolk colour	3.40	3.60	3.60	3.40	3.60	0.268	0.142	0.993	0.859
Shell thickness (µm)	19.5	19.8	21.1	20.1	20.5	0.549	0.691	0.163	0.288

*Note*: Means without common superscript (a and b) within a row are significantly different (*p < *0.05).

The effect of adding MM powder to the diet on egg quality traits of Japanese laying quails at 20 weeks of age is shown in Table 7. In contrast to the findings at 16 weeks, the egg‐shape index at 20 weeks of age was not significantly affected by dietary MM (*p* > 0.05). Furthermore, increasing MM powder levels from 0.2% to 0.8% of the diet did not result in significant linear or quadratic responses in any egg quality trait (*p* > 0.05) (Table 7).

**TABLE 7:** Effect of adding mountain mint (*Ziziphora clinopodioides)* to Japanese quails diet on egg quality traits at 20 weeks of age.

	Mountain mint (% of diet)					SEM	*p* value		
	0 (control)	0.2	0.4	0.6	0.8		Treatment	Linear	Quadratic
Specific gravity (g/cm^3^)	1.07	1.07	1.07	1.07	1.07	0.935	0.953	0.927	0.900
Albumen (%)	51.3	53.1	51.3	50.3	50.3	1.474	0.528	0.650	0.630
Yolk (%)	34.4	33.1	31.6	34.4	33.8	1.340	0.553	0.284	0.260
Shell (%)	14.3	13.8	14.9	15.3	13.6	0.648	0.534	0.352	0.335
Shape index (%)	79.1	78.3	78.1	77.5	76.2	0.994	0.336	0.664	0.096
Haugh unit	79.9	80.5	81.1	82.0	80.9	0.400	0.542	0.563	0.808
Yolk colour	3.40	3.21	3.60	3.01	3.60	0.253	0.401	0.611	0.547
Shell thickness (µm)	19.8	19.5	20.5	20.1	21.1	0.507	0.235	0.968	0.531

### 3.4 Blood Metabolites

The effects of adding MM aerial parts powder to the diet of laying Japanese quails from 9 to 20 weeks of age on blood metabolite concentrations and humoral immune response are presented in Tables 8 and 9, respectively. In this study, dietary MM significantly affected blood HDL‐C and LDL‐C concentrations in laying quails at 20 weeks of age. The highest HDL‐C concentration was observed in birds fed the diet containing 0.8% MM powder, which was significantly higher (*p* < 0.05) than that observed in the control and other treatment groups. Increasing MM powder levels from 0.2% to 0.8% resulted in a significant linear increase in blood HDL‐C concentration (*p* < 0.05); however, no significant quadratic trend was detected.

**TABLE 8:** Effect of adding mountain mint (*Ziziphora clinopodioides)* to laying Japanese quails diet on blood serum metabolites.

	Mountain mint (% of diet)					SEM	*p* value		
	0 (control)	0.2	0.4	0.6	0.8		Treatment	Linear	Quadratic
	Blood metabolites (mmol/L), 20 weeks of age								
TG	476.4	471.6	475.6	475.0	476.0	1.152	0.060	0.751	0.830
TC	165.0	160.2	164.0	164.2	164.4	2.513	0.070	0.284	0.260
HDL‐C	79.4^b^	78.0^b^	83.0^ab^	83.8^ab^	87.0^a^	1.200	0.001	0.185	0.584
LDL‐C	47.0^a^	43.8^b^	40.1^c^	40.0^c^	40.6^c^	1.065	0.041	0.089	0.071

*Note*: Means without common superscript (a–c) within a row are significantly different (*p* < 0.05).

Abbreviations: HDL‐C, high‐density lipoprotein cholesterol; LDL‐C, low‐density lipoprotein cholesterol; TC, total cholesterol; TG, triglyceride.

**TABLE 9:** Effect of adding mountain mint (*Ziziphora clinopodioides)* to laying Japanese quails diet on immune response.

	Mountain mint (% of diet)					SEM	*p* value		
	0 (control)	0.2	0.4	0.6	0.8		Treatment	Linear	Quadratic
	Antibody concentration response to SRBC inoculation (log_2_), 14 weeks of age								
Total‐anti SRBC	8.50	8.70	9.50	9.10	9.20	0.130	0.188	0.968	0.531
IgG	7.00	6.40	7.20	7.00	7.00	0.095	0.572	0.927	0.900
IgM	1.50^c^	2.30^a^	2.30^a^	2.10^b^	2.20^bc^	0.050	0.011	0.090	0.460

*Note*: Means without common superscript (a–c) within a row are significantly different (*p* < 0.05).

Abbreviations: IgG, immunoglobulin G; IgM, immunoglobulin M; SRBC, sheep red blood cells.

The lowest LDL‐C concentration was observed in birds fed diets containing 0.4%–0.8% MM powder, which was significantly lower (*p* < 0.05) than that of birds fed the control diet or the diet containing 0.2% MM. Increasing MM powder levels from 0.2% to 0.8% resulted in a nonsignificant linear decrease in blood LDL‐C concentration (*p* < 0.089), with no significant quadratic trend observed. Dietary MM supplementation had no significant effect on TG or TC concentrations, and no linear or quadratic trends were observed in response to increasing MM levels (*p* > 0.05) (Table 8).

### 3.5 Humoral Immune Response

As shown in Table 9, dietary MM significantly affected blood IgM concentration in laying Japanese quails at 14 weeks of age. Specifically, MM treatments increased IgM titres in response to SRBC compared with the control group (*p* < 0.05). The highest IgM titres were observed in birds fed diets containing 0.2% and 0.4% MM. In contrast, dietary MM supplementation had no significant effect on IgG or total anti‐SRBC titres (*p* > 0.05). No linear or quadratic trends were observed in antibody titres in response to increasing MM levels in the diet.

## 4 Discussion

### 4.1 Essential Oil Analysis of MM

The essential oil obtained from the aerial parts of MM exhibited a characteristic chemical profile dominated by monoterpenes, particularly oxygenated monoterpenes, as revealed by GC/MS analysis. Although 35 compounds were identified, only 10 major constituents accounted for 93.88% of the total oil, indicating a high degree of compositional concentration. Pulegone was the principal component (62.03%), followed by *cis*‐caran‐*trans*‐2‐ol (11.89%) and 1,8‐cineole (11.03%), whereas β‐pinene was present in lower amounts (2.04%). The predominance of pulegone is consistent with previous reports on *Ziziphora* species, in which pulegone‐rich chemotypes are commonly observed and considered a chemotaxonomic feature of the genus (Adams 2007; Safaii et al. 2022). Oxygenated monoterpenes such as pulegone and 1,8‐cineole are known for their pronounced biological activities, including antimicrobial, antioxidant and insecticidal effects, which may explain the traditional medicinal use of MM in folk medicine (Meral et al. 2002; Mazarei 2023). Variations in essential oil composition among different studies on *Ziziphora* are attributed to factors such as geographical origin, climatic conditions, plant part analysed, phenological stage and extraction method (Adams 2007; Safaii et al. 2022). The high content of oxygenated monoterpenes observed in the present study suggests that MM essential oil may possess considerable bioactive potential and supports further investigation into its pharmacological and industrial applications.

### 4.2 Productive Performance

Cellulose was used as an inert diluent and replaced by MM at low inclusion levels, which is a common practice in poultry feeding trials. Given the negligible digestibility of cellulose and the small amounts of nutrients contributed by MM at the inclusion rates used (≤0.8%), the experimental diets were formulated to remain nutritionally comparable. Therefore, the observed responses are more likely related to the biological activity of phytogenic compounds rather than differences in dietary nutrient content (Mateos et al. 2012).

The results of this study showed that adding MM powder to the diet of laying Japanese quails at different levels (0.2%–0.8% of the diet) did not have a significant effect on productive performance indicators. One possible reason for the lack of significant effects of adding MM powder on quail performance may be related to the selected doses. Although the doses used in this study appear appropriate, higher or lower concentrations than those investigated may be required to observe positive effects on productive performance. Additionally, it is possible that the active compounds present in MM powder were insufficient to stimulate productive performance in the birds.

Limited research has been conducted on the inclusion of MM in the diet of Japanese quails. Nobakht and Nabizade (2013) reported that the use of different levels of MM plant powder had significant effects on performance, egg quality traits and blood TG levels in laying hens. The highest values of EW, EM, FI, egg SG, albumen and egg production percentages, and the best FCR were obtained using 2% MM in the diet. The lowest blood TG levels were observed in birds fed diets containing 1.5% MM powder. They concluded that inclusion of 2% MM powder in laying hen diets improves performance and blood TG levels. However, in the present experiment, productive performance and egg quality traits—except for egg‐shape index, TGs and EW—were not affected. At the same time, adding MM to the diet of laying quails improved the blood lipid profile in this study.

Safamehr et al. (2012) reported that adding MM to the diet of broiler chickens at levels of 1.5% and 2% significantly increased FI, whereas supplementation with 1% MM reduced FCR compared to the control treatment. Supplementation with 1% MM also reduced abdominal fat percentage relative to the control. Adding MM had no significant effect on blood cholesterol, TG or glucose concentrations, nor on heterophil percentage or the heterophil‐to‐lymphocyte ratio. They concluded that dietary inclusion of 1% MM positively affects growth traits and immune response in broiler chickens. Conversely, Jaderi et al. (2011) reported that inclusion of MM in laying hen diets decreased EM and egg production.

Differences between the present findings in Japanese quails and the adverse effects on egg production reported by Jaderi et al. (2011), who used 2% MM in laying hen diets, may be explained by a combination of species‐specific physiology, dosage and differences in the chemical composition of MM. Japanese quails differ markedly from laying hens in metabolic rate, digestive efficiency and nutrient partitioning, which can influence their responsiveness to phytogenic feed additives. Previous studies have demonstrated that the effects of essential oils on egg production are highly dependent on bird species, age and production stage (Brenes and Roura 2010). In addition, the biological activity of MM is closely linked to its essential oil composition, which can vary widely depending on plant origin, harvest time and processing method, particularly regarding the relative proportions of monoterpenes such as pulegone, isomenthone and 1,8‐cineole (Sharifi‐Rad et al. 2017). At moderate inclusion levels, these compounds may enhance gut health, antioxidant status and nutrient utilization, thereby supporting egg production, whereas higher doses or different chemotypes may exert suppressive or toxic effects, as suggested in laying hen studies (Jaderi et al. 2011; Brenes and Roura 2010). Therefore, the contrasting results between the present study and those of Jaderi et al. (2011) likely reflect differences in species sensitivity, supplementation level and phytochemical profile of MM.

Similarly, Talebi et al. (2021) reported that dietary inclusion of MM extract significantly increased FI and daily weight gain and improved FCR in broiler chicks. Differences among studies may be attributed to variations in the form of supplementation (essential oils, extracts or plant powders) and the concentration of active compounds. The lack of growth‐promoting effects of herbal supplements in some studies may also be related to diet composition and environmental conditions; supplementation of MM in balanced diets under optimal environmental conditions may not affect production parameters. Genetic factors and environmental conditions can also play important roles in bird productive performance (Safamehr et al. 2012). Overall, although MM powder positively affected blood lipid profile and immune response in quails, its lack of significant impact on productive performance indicates that further detailed studies are needed to fully evaluate its effects. Such information may aid in developing more effective nutritional strategies to improve quail health and performance.

### 4.3 Egg Quality Traits

In the present study, egg quality traits were evaluated at 16 and 20 weeks of age to represent stable phases of the laying cycle in Japanese quails. During the early laying period, egg traits may fluctuate markedly due to rapid physiological adaptation, whereas measurements obtained at later stages more reliably reflect sustained dietary effects. Although assessment at earlier ages could provide additional information on initial post‐lay changes, the selected time points were considered appropriate for the objectives of this study. No specific a priori hypothesis regarding age‐dependent responses was established; however, egg quality was evaluated at two time points to capture potential temporal variation in the response to dietary MM.

The results showed that adding MM powder to the diet of laying Japanese quails at different doses (0.2%–0.8% of the diet) significantly affected only the egg‐shape index, which decreased linearly. No significant effects were observed on other egg quality traits, including egg SG, HU, egg yolk colour, albumen, yolk and shell percentages, or EST. Jaderi et al. (2011) similarly reported that MM inclusion in laying hen diets reduced eggshell weight and HU compared with control diets.

The egg‐shape index is an important indicator of egg quality and may influence hatchability and subsequent chick growth. EW is also correlated with egg‐shape index; however, this index alone does not determine hatchability. In Japanese quails, egg‐shape index, defined as the ratio of egg width to length, is primarily influenced by bird age and feather colour, although nutrition and health also play roles. Older quails tend to produce eggs with a higher shape index (rounder) due to age‐related changes in the oviduct and internal egg pressure, which affect egg dimensions (Zita et al. 2013). Differences in egg‐shape index among quails with different feather colours have also been reported, suggesting a genetic influence (Cahyadi et al. 2019). Environmental factors such as temperature, humidity, nutrition and health status further affect egg quality (Angel Daniel et al. 2022).

In the present experiment, birds were of the same age and maintained under identical environmental conditions, except for dietary treatments. Therefore, the observed decrease in egg‐shape index and increased egg elongation with higher MM inclusion levels likely reflect the effect of MM on the oviduct. These changes may be related to bioactive constituents of MM, particularly essential oil monoterpenes such as pulegone, isomenthone, 1,8‐cineole and piperitenone, which are known to modulate smooth muscle activity. Egg shape is largely determined during passage through the magnum and isthmus, where coordinated smooth muscle contractions and secretion of albumen and shell membranes occur. Alterations in oviductal tone or secretory activity can influence egg elongation and shape index. Monoterpenes have been reported to affect smooth muscle contractility and calcium signalling in visceral tissues, potentially modifying oviduct peristalsis and egg transit time. Prolonged or altered residence time in the oviduct has been associated with more elongated eggs in avian species (Pereira‐Gonçalves et al. 2018).

In the present study, a linear decrease in egg‐shape index was observed at 16 weeks of age, with eggs becoming less round as MM levels increased; however, this effect was not significant at 20 weeks of age. González (1995) reported that egg‐shape index in Japanese quails decreased significantly between early laying ages (8–17 weeks) but stabilized at later ages, indicating maturation‐related stabilization of egg morphology. Thus, the observed effect at 16 weeks may reflect an interaction between dietary bioactive compounds in MM and ongoing reproductive system development during early lay. By 20 weeks of age, reproductive and eggshell formation processes may be sufficiently stabilized to attenuate dietary effects on egg‐shape index.

### 4.4 Blood Metabolites

The results of this study showed that adding MM powder to the diet of laying Japanese quails at different doses (0.2%–0.8% of the diet) positively affected the blood lipid profile of the birds. In particular, the increase in HDL‐C and the decrease in LDL‐C levels in the quail blood indicate the positive effects of this medicinal plant on the cardiovascular health of consumers. HDL‐C is known as ‘good’ cholesterol because it helps remove excess cholesterol from the bloodstream and transport it to the liver for metabolism. Increasing HDL‐C levels can help reduce the risk of cardiovascular diseases. Conversely, LDL‐C is known as ‘bad’ cholesterol, and increased levels are usually associated with a higher risk of heart disease. The reduction in LDL‐C levels in quails may help improve the overall health of consumers and reduce the risk of metabolic diseases (Alagawany et al. 2021).

The lack of changes in TG and TC concentrations is also noteworthy. This may indicate that the positive effects of MM powder are focused more on lipid distribution and composition rather than on total lipid content. These findings suggest that MM may activate specific mechanisms that improve lipid balance without affecting overall lipid levels. Active compounds in MM powder, such as flavonoids and phenolic compounds, may play an important role in these effects. These compounds possess antioxidant and anti‐inflammatory properties that may enhance metabolic function and regulate lipid metabolism. They may also improve blood lipid profiles by affecting enzymes related to lipid metabolism and liver function (Abd El‐Hack et al. 2025).

These findings may have important practical applications in the quail farming industry. The use of natural additives such as MM may have potential to improve certain aspects of metabolic health in birds; however, the practical significance of these effects requires further investigation. This approach may also reduce the need for chemical supplements and synthetic drugs, thereby increasing the quality of livestock products and improving overall public health.

The lipid‐lowering effects of some medicinal plants, their essential oils or their major bioactive compounds have been previously reported (Montazeri et al. 2014). However, few studies have focused on the effects of essential oils on lipid metabolism in poultry. Essential oils and some of their active constituents can reduce plasma cholesterol and TG levels, possibly through inhibition of enzymes involved in lipid synthesis by monoterpenes. The cholesterol‐lowering effect of essential oils has been attributed to inhibition of the hepatic enzyme 3‐hydroxy‐3‐methylglutaryl‐CoA reductase (HMG‐CoA reductase), a key regulatory enzyme in cholesterol synthesis (Elson 1995). Major bioactive constituents of MM include pulegone, isomenthone, 1,8‐cineole and piperitenone. These monoterpenes are known to exert hypolipidemic effects through modulation of lipid metabolism, particularly via inhibition of HMG‐CoA reductase activity, which reduces endogenous cholesterol synthesis. In addition, compounds such as 1,8‐cineole and isomenthone have been reported to influence hepatic lipid regulation and improve serum lipid profiles through antioxidant and enzyme‐modulating mechanisms (Bahr et al. 2021). Beyond possible HMG‐CoA reductase inhibition, the observed rise in HDL‐C and fall in LDL‐C suggests MM compounds may regulate lipoprotein metabolism through non‐classical pathways. Monoterpenes like pulegone and 1,8‐cineole can influence hepatic lipid handling, apolipoprotein expression and lipoprotein assembly, potentially enhancing reverse cholesterol transport without altering TC or TGs. However, the use of MM powder in the diet of laying hens did not significantly affect total protein, TG, cholesterol, albumin, glucose or immune cell counts (Jaderi et al. 2011).

In the present experiment, TG and total blood cholesterol concentrations were not affected by different levels of dietary MM. Similarly, Demir et al. (2003) reported that supplementation with several medicinal plant powders (thyme, oregano, garlic and cinnamon) had no significant effect on blood TG concentrations in broiler chicks. In contrast, Genedy (2003) observed significant reductions in serum cholesterol and total lipid concentrations in Japanese quails fed chamomile and thyme, indicating that lipid responses may vary depending on plant species and bioactive composition. Differences between the present results and those of Genedy (2003) likely reflect variations in phytochemical profiles and experimental conditions. MM contains mainly pulegone and cineole, whereas thyme and chamomile are richer in phenolic monoterpenes and flavonoids linked to stronger inhibition of hepatic cholesterol synthesis. As a result, MM may affect lipoprotein distribution and reverse cholesterol transport rather than TC levels. Variations in dosage, supplementation duration, bird genotype and physiological stage may also explain inconsistent lipid responses across studies.

### 4.5 Humoral Immune Response

The results of this study showed that adding MM to the diet of laying Japanese quails positively affected IgM antibody titres in laying Japanese quails, whereas no effects were observed on IgG or total immunoglobulin concentrations in the blood. IgM is the first antibody produced in response to infection and represents the primary immune response. An increase in IgM titre may indicate stimulation of the primary immune response and activation of host defence mechanisms against pathogens (Mehrzad et al. 2024). This effect may be attributed to active compounds present in MM that act as immunostimulants. Phenolic and flavonoid compounds in MM may enhance antibody production by increasing the activity of immune cells such as lymphocytes and macrophages. In contrast, the absence of changes in IgG and total immunoglobulin levels suggests that the secondary immune response and immune memory were not affected. IgG is typically produced during later stages of infection and reflects a more sustained immune response (Mehrzad et al. 2024). These findings suggest that MM supplementation may primarily stimulate early immune responses, and further investigation is required to evaluate its long‐term effects on avian immunity. The results of this study have practical implications for the quail industry, as the use of natural additives such as MM may serve as an effective strategy to enhance immune function and reduce reliance on antibiotics and chemical drugs. Given growing concerns about antibiotic resistance, natural alternatives represent an attractive option.

In the present study, humoral immune response was evaluated using the SRBC assay at a single time point, a widely accepted method for assessing antibody‐mediated immunity in poultry following exposure to a non‐pathogenic, T‐cell‐dependent antigen. Sampling at a fixed time point after SRBC injection is commonly used to capture a representative primary antibody response for comparison among dietary treatments (Maghsoudi et al. 2020). The timing of SRBC administration at 14 weeks of age coincided with a stable physiological and nutritional status during the laying period. Nevertheless, a single‐point measurement does not fully reflect the kinetics or persistence of humoral immune responses, as antibody titres can vary over time. Future studies incorporating multiple post‐immunization sampling points would provide a more comprehensive evaluation of immune response dynamics.

## 5 Conclusions

Dietary inclusion of MM in laying Japanese quail diets did not improve production performance; however, it did not compromise this important parameter. Egg‐shape index decreased with increasing MM inclusion levels, resulting in more elongated eggs, which may have implications for egg quality and warrants further investigation into the factors affecting egg morphology. MM supplementation positively affected blood lipid profiles and immune responses in quails. These findings suggest that adding MM to quail diets may benefit both consumer and bird health. The favourable effects of MM on lipid metabolism and immune function highlight its potential as a valuable dietary supplement, although further research is needed to assess the long‐term consequences of MM supplementation.

## Author Contributions

**Ahmad Hassanabadi**: project administration, software, methodology, conceptualization, funding acquisition, writing – review and editing. **Mojtaba Teymoori Oghaz**: investigation, data curation, software, formal analysis. **Heydar Zarghi**: advising, review the manuscript.

## Funding

This research was supported by the Ferdowsi University of Mashhad [project number 54800].

## Ethics Statement

The authors confirm that the ethical policies of the journal, as noted in the journal's author guidelines page, have been adhered to, and the appropriate ethical review committee approval has been received. The authors confirm that they have followed EU standards for the protection of animals used for scientific purposes and feed legislation. Moreover, this study was conducted according to the procedures established by the Iranian Ministry of Agriculture (Experimental Authorization No. ASRI‐2016‐95014).

## Conflicts of Interest

The authors declare no conflicts of interest.

## Data Availability

The data that support the findings of this study are available from the corresponding author upon reasonable request.
